# Higher Concentrations of BCAAs and 3-HIB Are Associated with Insulin Resistance in the Transition from Gestational Diabetes to Type 2 Diabetes

**DOI:** 10.1155/2018/4207067

**Published:** 2018-06-05

**Authors:** Ulrika Andersson-Hall, Carolina Gustavsson, Anders Pedersen, Daniel Malmodin, Louise Joelsson, Agneta Holmäng

**Affiliations:** ^1^Institute of Neuroscience and Physiology, Sahlgrenska Academy, University of Gothenburg, Gothenburg, Sweden; ^2^Swedish NMR Centre, University of Gothenburg, Gothenburg, Sweden

## Abstract

**Aim:**

Determine the metabolic profile and identify risk factors of women transitioning from gestational diabetes mellitus (GDM) to type 2 diabetes mellitus (T2DM).

**Methods:**

237 women diagnosed with GDM underwent an oral glucose tolerance test (OGTT), anthropometrics assessment, and completed lifestyle questionnaires six years after pregnancy. Blood was analysed for clinical variables (e.g., insulin, glucose, HbA1c, adiponectin, leptin, and lipid levels) and NMR metabolomics. Based on the OGTT, women were divided into three groups: normal glucose tolerance (NGT), impaired glucose tolerance (IGT), and T2DM.

**Results:**

Six years after GDM, 19% of subjects had T2DM and 19% IGT. After BMI adjustment, the IGT group had lower HDL, higher leptin, and higher free fatty acid (FFA) levels, and the T2DM group higher triglyceride, FFA, and C-reactive protein levels than the NGT group. IGT and T2DM groups reported lower physical activity. NMR measurements revealed that levels of branched-chain amino acids (BCAAs) and the valine metabolite 3-hydroxyisobyturate were higher in T2DM and IGT groups and correlated with measures of insulin resistance and lipid metabolism.

**Conclusion:**

In addition to well-known clinical risk factors, BCAAs and 3-hydroxyisobyturate are potential markers to be evaluated as predictors of metabolic risk after pregnancy complicated by GDM.

## 1. Introduction

Gestational diabetes mellitus (GDM), defined as glucose intolerance with onset or first recognition during pregnancy [1], occurs in up to 14% of pregnancies every year [2]. Although in most women glucose tolerance returns to normal after delivery, the frequency of GDM correlates with the prevalence of type 2 diabetes mellitus (T2DM), and women with GDM have increased risk for T2DM later in life. The 10-year risk is ~40%, and the incidence is highest in the first 5 years after pregnancy [3]. In both GDM and T2DM, chronic insulin resistance and pancreatic *β*-cell dysfunction play crucial pathogenic roles. Pregnancy, an inherently insulin-resistant state, may reveal a preexisting deficiency in insulin secretion and insulin sensitivity and relative *β*-cell failure. Thus, the stress of pregnancy may reveal a predisposition to T2DM and provide early signs useful for preventing chronic diseases.

Comprehensive metabolic profiling (metabolomics) has provided unique insights into the mechanisms of insulin resistance. For example, branched-chain amino acids (BCAAs) and the aromatic amino acids (AAAs) are strongly associated with metabolic disease [4]. The BCAA-related metabolite cluster is tightly associated with the incidence of T2DM in cross-sectional and case-control studies and across multiple ethnic groups and geographies. Altered flux through the BCAA catabolic pathway, rather than BCAA concentrations per se, appears to be especially important. Considerable evidence implicates free fatty acids (FFAs) and other lipids that contribute to metabolic disease and insulin resistance. These results raise the possibility that lipids and BCAAs and interactions between BCAAs, FFAs, and glucose oxidative pathways have combined effects [5]. Metabolomics has potential prognostic power and may provide insights into the mechanisms of insulin resistance and T2DM.

Recently, we characterized the metabolic transition profile from pregnancy to postpartum by mass spectrometry-based metabolomics in women with GDM [6]. These women had significantly higher postpartum levels of BCAAs (especially valine) than women with healthy pregnancies, and their circulating lipids did not return to normal levels after pregnancy. BCAAs were not elevated during pregnancy and therefore increased postpartum.

3-Hydroxy-isobutyrate (3-HIB), a catabolic intermediate of the BCAA valine, may be released from muscle cells and regulates transendothelial transport and muscle cell uptake of FFAs [7, 8]. Thus, 3-HIB may be a signalling metabolite that connects the regulation of FFA flux to BCAA catabolism. This novel hypothesis might explain how increased BCAA levels cause insulin resistance and diabetes.

In this study, we compared the metabolic profiles of women who had impaired glucose tolerance (IGT) or T2DM or were normoglycaemic 6 years after GDM. We hypothesized that alterations in BCAA metabolism interact with lipid metabolism and glucose oxidative pathways to induce insulin resistance and diabetes.

## 2. Methods

### 2.1. Subjects

**Figure 1:**
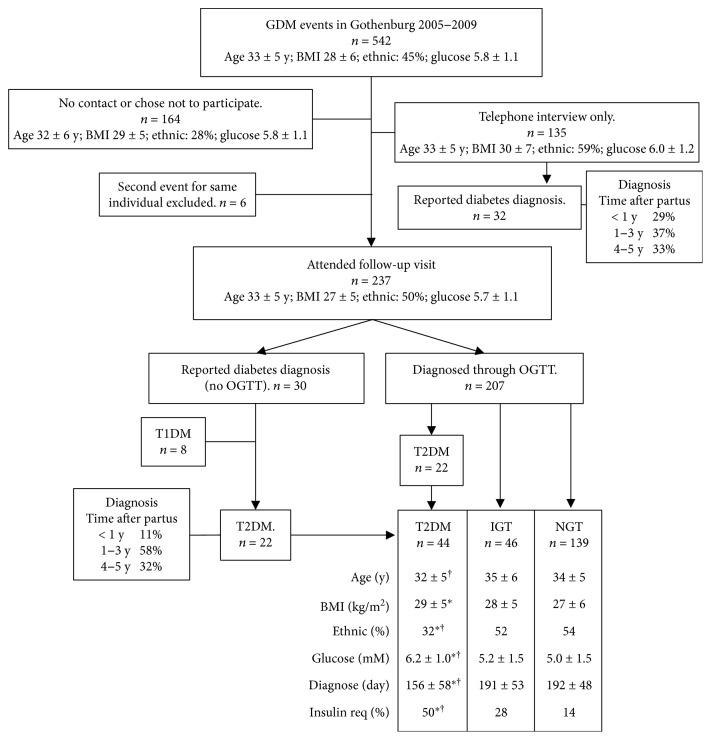
Flow chart of study population and pregnancy data. Age, age at partus; BMI, BMI at start of pregnancy (kg/m^2^); ethnic, % Nordic ethnicity; glucose, fasting p-glucose (mM) at time of GDM diagnosis; diagnose, gestational age at time of GDM diagnosis; and insulin req, required insulin treatment during GDM pregnancy. ^∗^ *P* < 0.05 versus NGT. ^†^ *P* < 0.05 versus IGT. Values are mean ± SD.

The study was approved by the ethics committee at the University of Gothenburg (402-08/750-15). Informed consent was obtained from all participants. Data were collected retrospectively from women in the Gothenburg area who were diagnosed with GDM from 2005 to 2009 (*N* = 542). Upon contact, women were invited to participate in the study. 378 women were briefly interviewed by telephone and asked to come to a follow-up visit. 237 women completed this visit at 5.6 ± 0.5 years after pregnancy (Figure 1). Follow-up participants were less frequently of non-Nordic ethnicity (*P* < 0.001) and had lower pregestational body mass index (BMI) and a lower fasting glucose value at GDM diagnosis (*P* < 0.01) than women who could not be reached or chose not to participate. If a woman had GDM during two pregnancies during the study interval, only data from the first pregnancy and follow-up were analysed (Figure 1).

### 2.2. Protocol

At follow-up, fasting blood samples were collected for all women, and those who had not previously been diagnosed with diabetes or who did not have a diagnostic fasting plasma glucose value underwent a 2 h 75 g OGTT. Venous blood for analysis of plasma glucose and serum insulin was collected at 0, 30, 60, 90, and 120 minutes. Capillary glucose was measured at 0 and 120 minutes as a backup in case venous sampling was unsuccessful. Fasting serum and plasma blood samples were used for further analysis.

Anthropometric measurements (weight, height, waist, and hip circumference) and resting blood pressure were determined, and participants were asked to complete questionnaires during the visit. A self-administered dietary questionnaire was used to assess energy intake (EI) during the 3 previous months [9]. The questionnaire has a semiquantitative food frequency design and was validated in Swedish men and women against a 4-day food record and 24 h energy expenditure and nitrogen excretion [9]. Basal metabolic rate (BMR) was calculated with Henry's equation [10]; subjects with an EI/BMR ratio < 0.95 or >3.0 were excluded. Physical activity was assessed by asking participants to rate the activity level between 0 and 4 at work and 1 and 4 at leisure with clear description of each level. To create an overview of activity level, reported activities at leisure and work were added to give a maximum of 8. Only fully and correctly completed questionnaires were used for analysis. In a subgroup of women (*n* = 87), fat mass, fat-free mass and body fat % was measured with air displacement plethysmography (BOD POD, software version 5.4.0, COSMED) at a second visit. Data from the GDM pregnancy were collected from medical records. BMI at the start of pregnancy was calculated from the weight recorded at the first antenatal visit performed during gestational weeks 6–12 and BMI change as the difference between BMI at follow-up and BMI at the start of pregnancy.

### 2.3. Glucose Tolerance Groups

Based on fasting and 2 h glucose values from the OGTT at the 6-year follow-up, three groups were created: normal glucose tolerance (NGT), IGT (including impaired fasting glucose or impaired glucose tolerance), and T2DM (including subjects with previously diagnosed T2DM). Classification was based on venous glucose levels according to the 1999 WHO guidelines [11]. In those subjects where venous glucose levels were not obtained, capillary glucose measurements were used to calculate corresponding venous values as described [12]. These values are only used for diagnosis and are not included in the analysis. Individuals with type 1 DM (T1DM) at follow-up, confirmed with antibody tests (GADA and IA-2A), were excluded from the main analysis but were included in a subgroup analysis of ketone metabolism.

### 2.4. Biochemical Measurements

HbA1c was analysed immediately with a point-of-care analyser (Afinion AS100; Axis-Shield, Oslo, Norway). Glucose, insulin, cholesterol (total, low-density lipoprotein (LDL), high-density lipoprotein (HDL)), triglycerides, leptin, adiponectin, C-reactive protein, and FFAs were analysed at the accredited Clinical Chemistry Laboratory, Sahlgrenska University Hospital (International Standard ISO 15189:2007). ELISA was used to measure adiponectin (Human Adiponectin ELISA kit, Millipore, Billerica, MA; interassay coefficient of variation, 7.0% at 10.5 mg/L) and leptin (Human Leptin Quantikine, R&D Systems; interassay coefficient of variation, 8.0% at 9 *μ*g/L). All other assays were routinely done with a cobas modular system (Roche Diagnostics). Homeostatic model assessment of insulin resistance (HOMA-IR) was calculated as (fasting glucose × fasting insulin)/22.5 [13]. Adipose tissue insulin resistance (ADIPO-IR) was calculated as fasting FFA × fasting insulin [14]. Due to occasional technical problems either with blood sampling or blood analysis, full sets for analysis were not available for all parameters at all time points. See Table 1 for *n* numbers.

### 2.5. NMR Spectroscopy

224 fasting serum samples were available for NMR analysis after all other blood analysis was performed. NMR data were acquired on a Bruker 800 MHz spectrometer equipped with a 3 mm TCI CryoProbe and a cooled SampleJet sample changer using the “zgespe” pulse sequence encoding a one-dimensional ^1^H experiment with suppression of water and J-modulation by excitation sculpting and perfect echo, respectively, and T2 filtering with a CPMG pulse train. For each sample, 128 scans were collected into 65k points with an acquisition time of 2.04 seconds, relaxation delay of 3 seconds, sweep width of 20 ppm, and a total CPMG pulse train of 193 ms (total experimental time, 12 minutes 4 seconds). The temperature was kept at 6°C in the sample changer and at 25°C during acquisition. Spectral data were processed with TopSpin 3.5pl6 (Bruker BioSpin), zero filling once, and 0.3 Hz line broadening before Fourier transform. Representative samples for the different groups were subjected to two-dimensional NMR data acquisition using ^1^H-^1^H TOCSY (“mlevgpphw5”), ^1^H,^13^C-HSQC (“hsqcetgpsisp2.2”), and two-dimensional Jres (“jresgpprqf”) experiments. Two-dimensional results for 3-HIB are shown in Supplementary Materials (available here). Experimental details are available on request. Processed one-dimensional data were imported into MATLAB 2016a (MathWorks) and aligned with icoshift [15], and peaks were integrated with an in-house MATLAB routine. NMR integrals were expressed as a percentage of NGT. Two-dimensional data, the human metabolome database [16] and Chenomx 8.3 (Chenomx Inc.) were used to annotate metabolite peaks.

### 2.6. Statistical Analysis

Pregnancy data were analysed with one-way ANOVA and post hoc Tukey analysis or chi-square test (binary variables). Follow-up measurements were analysed with ANOVA and ANCOVA (BMI as covariate). To predict the likelihood of T2DM from pregnancy data, variables were analysed by binary regression (outcome T2DM, yes/no). For association of follow-up variables with insulin resistance, a stepwise forward linear regression model was built with HOMA-IR as the dependant variable. All variables in Table 1 that are not direct measures of glucose metabolism (glucose, insulin, and HbA1c) plus metabolites from Table 2 involved in fat and amino acid metabolism were entered as predictors. Ketone bodies did not correlate with HOMA-IR and were excluded. Bivariate and partial correlations (BMI as control variable) were further performed. *P* < 0.05 was considered significant. All values are mean ± SD unless otherwise stated.

## 3. Results

### 3.1. Risk Factors for T2DM 6 Years after GDM

After the follow-up OGTT, 58% had NGT (*n* = 139), 19% IGT (*n* = 46), 19% T2DM (*n* = 44), and 3% T1DM (*n* = 8). In the T2DM group, BMI at start of pregnancy was higher than in the NGT group (Figure 1), GDM was diagnosed earlier, fasting glucose was higher at GDM diagnosis, and higher percentages required insulin during pregnancy or were non-Nordic.

By logistic regression, the strongest prediction model for development of T2DM included fasting glucose at GDM diagnosis (*P* = 0.001, Exp(B) = 1.92), gestational age at diagnosis (*P* = 0.004, Exp(B) = 0.99), and insulin treatment (*P* = 0.03 Exp(B) = 2.5). These factors predicted 81% of T2DM cases 6 years later.

### 3.2. Clinical Characteristics and Physical Activity

Anthropometric and clinical data at the 6-year follow-up are shown in Table 1. Women with IGT or T2DM had higher BMI than normoglycaemic women, but only in the IGT group did BMI increase from prepregnancy to follow-up (*P* < 0.001). Anthropometric measurements were all higher in the IGT and T2DM groups than in the NGT group.

As expected, glucose, insulin, and HOMA-IR were higher in the IGT and T2DM groups even after BMI adjustment. HbA1c was significantly increased in T2DM only. HDL was lower and leptin was higher in the IGT and T2DM groups than in the NGT group, but only the differences between IGT and NGT were significant after BMI adjustment. FFA and ADIPO-IR, reflecting inability of insulin to suppress peripheral lipolysis, were higher in the IGT and T2DM groups, and the difference was highly significant even after BMI adjustment; there were no differences in total cholesterol or LDL. The leptin : adiponectin ratio and C-reactive protein (CRP) were both higher in T2DM, independent of BMI. Physical activity was higher in the NGT group than in the other groups, even after BMI adjustment, and correlated with several metabolic parameters (BMI, waist circumference, hip circumference, HbA1c, fasting insulin, 2-hour OGTT glucose, HOMA-IR, HDL, leptin, ADIPO-IR, and CRP). After adjustment for BMI, significant correlations remained for hip circumference (*r* = −0.22, *P* = 0.003), HOMA-IR (*r* = −0.23, *P* = 0.002), ADIPO-IR (*r* = −0.20, *P* = 0.007), and CRP (*r* = −0.20, *P* = 0.005). Self-reported dietary intake did not differ in the groups.

### 3.3. Metabolomics

**Figure 2:**
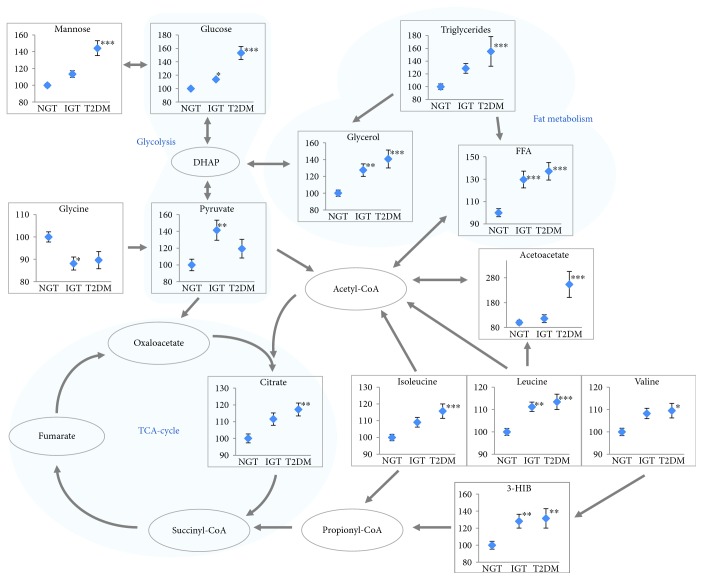
Map of metabolites that differed significantly between groups after BMI adjustment 6 years after a GDM pregnancy. Values are percent of NGT (mean ± SEM). *n* = 137 for NGT, *n* = 43 for IGT, and *n* = 44 for T2DM. ^∗^ *P* < 0.05, ^∗∗^ *P* < 0.01, and ^∗∗∗^ *P* < 0.001 versus NGT by ANCOVA with BMI as covariate.

NMR metabolomics of serum samples from the 6-year follow-up showed significant differences between groups (Table 2). Metabolites that differed significantly after BMI adjustment are shown in Figure 2. All three BCAAs were significantly increased in the T2DM group after BMI adjustment, and there was a tendency to increase for all BCAAs in the IGT group, but only the increase in leucine was significant. 3-HIB increased by ~30% in both the IGT and T2DM groups. AAA concentrations were significantly elevated in IGT (Tyr) and in T2DM (Phe) by ANOVA but not after BMI adjustment.

**Figure 3:**
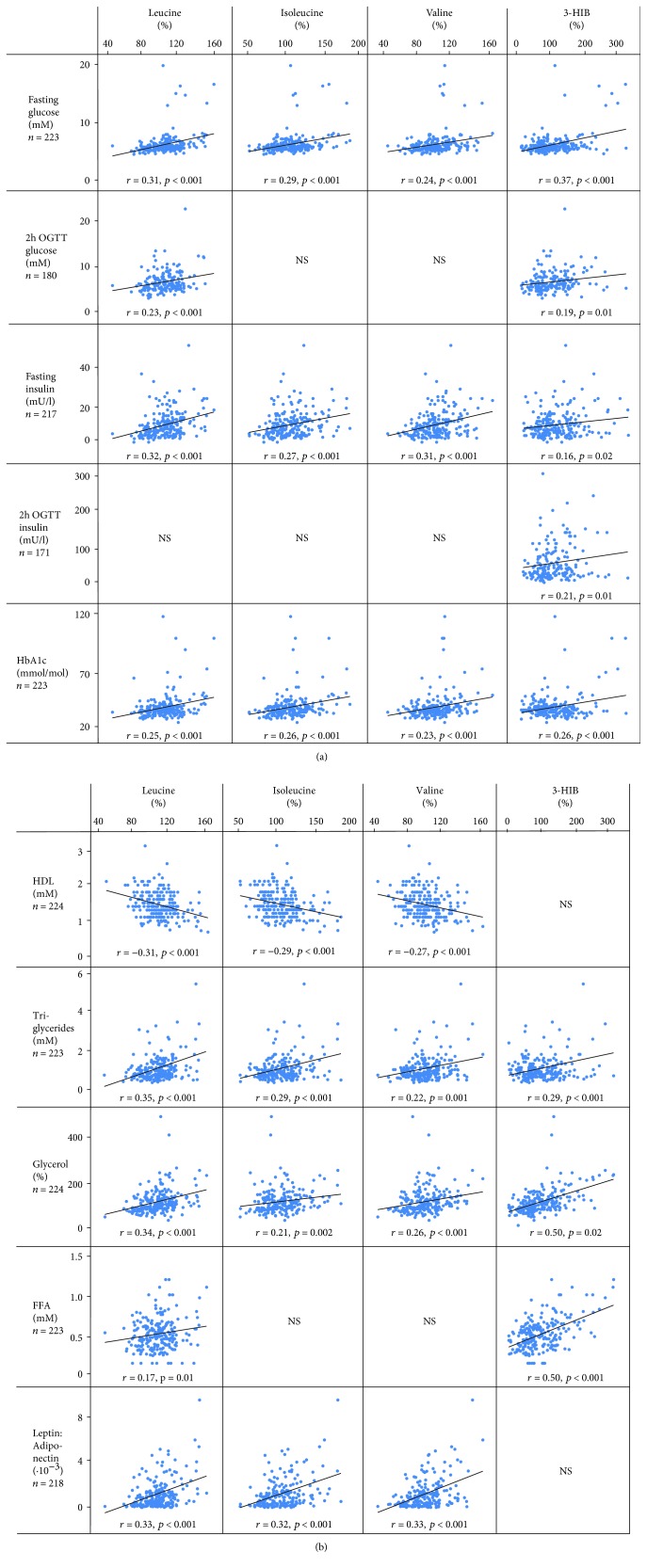
Correlations between glucose (a) and lipid (b) metabolism measurements and BCAAs, 3-HIB, and AAAs 6 years after GDM pregnancy. All three groups (NGT, IGT, and T2DM) were included. Only correlations that were significant after BMI adjustment are shown (*P* < 0.05).

Figure 3 shows correlations of BCAAs and 3-HIB with glucose and lipid metabolism measurements that remained significant after BMI was introduced as a covariate. 3-HIB correlated with all glucose and insulin measurements, whereas BCAAs mainly correlated with fasting values (Figure 3(a)). For lipid metabolites and adipokines, 3-HIB correlated only with triglycerides and its breakdown/precursor metabolites glycerol and FFA. All BCAAs correlated positively with triglycerides, glycerol, and the leptin : adiponectin ratio and inversely with HDL. Of the BCAAs and 3-HIB, only leucine correlated with body fat percentage (*P* < 0.01, *R* = 0.3, *n* = 89).

For AAAs, Tyr correlated with fasting insulin (*P* < 0.001, *R* = 0.31) and the leptin : adiponectin ratio (*P* < 0.001, *R* = 0.28), whereas Phe did not correlate with any glucose metabolism or lipid parameters after BMI adjustment.

Other group differences included for T2DM increased mannose, acetoacetate, glycerol, and citrate and for IGT increased pyruvate and glycerol and decreased glycine.

### 3.4. Regression Model to Predict HOMA-IR

To predict HOMA-IR variance, follow-up variables that correlated with HOMA-IR but are not direct measurements of glucose metabolism were analysed in a regression model. These included BMI, BMI change, age, blood pressure, waist and hip circumference, waist : hip ratio, triglycerides, cholesterol, HDL, LDL, CRP, leptin, adiponectin, leptin : adiponectin ratio, FFA, phenylalanine, tyrosine, glycine, valine, leucine, isoleucine, 3-HIB, and physical activity. After stepwise analysis, the model contained eight variables that gave an adjusted *R* ^2^ = 0.62 (Table 3). The three variables with the highest contribution to explain HOMA-IR variance were triglycerides (*β* = 0.35, *P* < 0.001), waist circumference (*β* = 0.31, *P* < 0.001), and 3-HIB (*β* = 0.19, *P* < 0.001). Other contributing variables were age, serum CRP, diastolic blood pressure, physical activity, and BMI change.

### 3.5. T1DM and Ketone Metabolism

**Figure 4:**
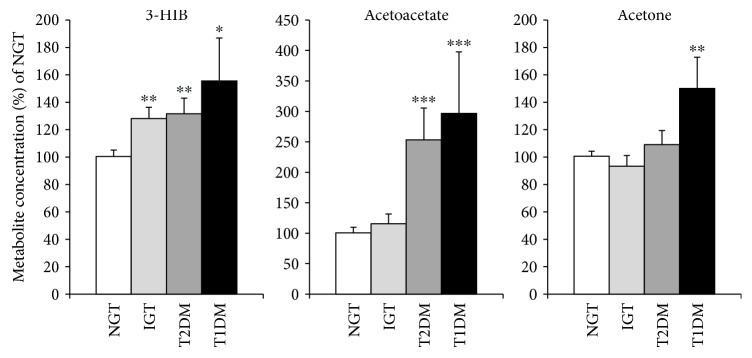
3-HIB and ketone concentrations in T1DM (*n* = 8), T2DM (*n* = 44), and IGT (*n* = 43) versus NGT (*n* = 137). ^∗^ *P* < 0.05, ^∗∗^ *P* < 0.01, and ^∗∗∗^ *P* < 0.001 versus NGT by ANCOVA with BMI as covariate.

Eight women were diagnosed with T1DM 6 years after GDM. This group had higher levels of acetoacetate and acetate than the NGT group (Figure 4) and the highest level of 3-HIB; no differences in BCAAs were found (data not shown). For the whole population, 3-HIB correlated with acetoacetate (*P* < 0.001, *R* = 0.386, *n* = 232).

## 4. Discussion

This study shows that the metabolic profile in women with IGT or T2DM differs substantially from that of women with NGT 6 years after GDM. At follow-up, 22% of the women had developed diabetes (19% T2DM and 3% T1DM) and 19% had IGT. Women with IGT and diabetes had an increased clinical risk profile (higher fasting glucose and insulin treatment during pregnancy) and developed diabetes at an earlier gestational age than those who remained normoglycaemic after pregnancy. These clinical risk factors, which have been shown in other follow-up studies [17], predicted 81% of T2DM cases 6 years after GDM pregnancy.

The IGT and T2DM groups had significant differences in amino acids. AAAs and BCAAs were increased and glycine was decreased. The increases for T2DM in leucine, isoleucine, valine, and 3-HIB were significant after BMI adjustment, but those in tyrosine and phenylalanine were not. The BMI-independent differences in precursors and intermediates such as mannose, glucose, glycine, pyruvate, and citrate point to a possible dysregulation of glycolysis and the TCA-cycle. Changes in triglycerides, glycerol, and FFA suggest dysfunctional fat metabolism. In regression models, the risk factors mainly associated with HOMA-IR are in order of importance triglycerides, waist circumference, 3-HIB, age, CRP, diastolic blood pressure, physical activity, and change in BMI from pregnancy to follow-up.

Disturbed AA metabolism has long been considered a feature of obesity and associated metabolic disease; obese people have higher blood levels of the BCAAs (leucine, isoleucine, and valine) and AAA (phenylalanine and tyrosine) but lower levels of glycine than lean people [18]. We obtained similar results but after BMI adjustment, some of these significances disappeared, linking the metabolism of BCAAs, triglycerides/fatty acids cycle, and glycolysis more strongly to the reduced insulin sensitivity and increased risk of IGT and T2DM per se. Only leucine correlated with body fat percentage. Levels of leucine, isoleucine, and valine are increased as early as 7 years before T2DM development [19]. In one study, BCAAs were elevated 6–9 weeks postpartum in GDM women who were at highest risk for T2DM [20]. Evidently, this metabolic profile precedes the onset of disease rather than being a consequence of T2DM.

BCAAs differ from other AAs in that they are initially catabolized primarily in skeletal muscle not in liver [21]. Indeed, ~50% of skeletal muscle AA uptake consists of BCAAs, since leucine, isoleucine, and valine avoid the hepatic metabolism of substrates immediately after absorption from the intestine [22]. Complete catabolism of BCAAs requires several enzymatic steps. As with most AAs, the final step results in acetyl-CoA, propionyl-CoA, and succinyl-CoA formed from catabolism of isoleucine, leucine, valine, methionine, citrate, and fatty acids—all of which were significantly higher in T2DM than NGT subjects in our study. While some reactions are reversible, leucine, isoleucine, and valine cannot be resynthesized. The catabolic pathways of these BCAAs share two catabolic enzymes in the first step; thereafter, the pathways diverge. The ketogenic leucine product, isovaleryl-CoA, is finally catabolized to acetyl-CoA and acetoacetate. Both valine and isoleucine are considered glucogenic, being metabolized to propionyl-CoA and acetyl-CoA, respectively.

One important step in valine metabolism is generation of 3-HIB, which can be released from tissues. Elevated plasma concentrations of 3-HIB in 3-day fasted subjects and in T1DM patients fasted overnight reflect augmented valine degradation from net body protein breakdown [23]. Since 3-HIB can serve as a gluconeogenic substrate [24], elevated plasma 3-HIB may have a physiologic function in fasting. The increased postabsorptive plasma 3-HIB levels in our subjects with diabetes may reflect increased protein breakdown as a consequence of relative insulin deficiency due to insufficient insulin secretion in relation to insulin resistance, which would also explain increased ketone body production [25]. The tight connection to fasting is also shown by the strong correlation between 3-HIB and both fasting glucose and insulin. Acetoacetate and 3-HIB were correlated and were elevated in both T1DM and T2DM women, whereas only 3-HIB was elevated in the IGT group. The high 3-HIB levels in the circulation and in skeletal muscle in diabetic subjects may lead to excess FFA uptake into muscle [7] and to incomplete lipid degradation [26], resulting in impaired fat oxidation and accumulation of lipotoxic intermediates, which adds to the metabolic inflexibility, impaired insulin signalling, and insulin resistance [7]. Consistent with these findings, 3-HIB had a strong linear correlation with triglycerides, glycerol, FFA, and ADIPO-IR. Interestingly, 3-HIB and body fat were not correlated. The tight relationship of BCAAs to metabolism in skeletal muscle, the most important tissue for insulin resistance, might also explain the strong correlations between 3-HIB and 2 h OGTT glucose and insulin, HOMA-IR, and HbA1c that we found.

Increased BCAA and 3-HIB levels can either be explained by increased protein breakdown and/or decreased catabolism of BCAAs and 3-HIB. In a recent study, several genes involved in BCAA catabolism were downregulated in muscle from insulin-resistant subjects [26]; the highest correlation was seen with *MUT* and *ALDH6A1*, both involved in valine catabolism downstream of 3-HIB. Consistent with this finding, we found a 10–15% increase in BCAAs but a 30% increase in HIB, suggesting 3-HIB accumulation due to impaired catabolism. Experimentally induced defects in MUT reduced cellular respiration and lipid oxidation, eventually leading to lipid accumulation in muscle and insulin resistance [26]. These results together with our findings point to BCAAs, particularly 3-HIB, as a sensitive and early marker for impaired glucose and lipid metabolism.

American Diabetes Association guidelines for postpartum glucose testing were revised in 2017 to include a 75 g OGTT at 4–12 weeks postpartum and thereafter every 1–3 years for women with a prior GDM diagnosis, and more frequent testing if screening results fall within the prediabetes ranges [27]. Low rates of postpartum glucose testing after GDM have been reported for several cohorts of women, despite national guidelines requiring glucose monitoring, and transition to primary care; fewer than half of women with GDM receive postpartum glucose testing [28]. In our study, all women visited the maternal clinic 6–8 weeks postpartum for a 3-day self-monitored glucose profile and a nonfasting glucose sample. They were then given a medical referral and advised to contact primary care for annual follow-up starting 1 year postpartum. Nevertheless, only 42% of our women visited a primary care physician for aftercare during the study period, confirming the reported low rates of follow-up with glucose testing and primary care visits after delivery [28]. Possible reasons for the low follow-up rate include logistical difficulties in administering an OGTT, a time-consuming and inconvenient procedure, fear of receiving a diagnosis of diabetes, and failure to attend the postpartum follow-up examination [20].

Pharmaceutical intervention and lifestyle modifications are as effective in delaying or preventing the onset of T2DM after a GDM pregnancy as they are in other cases of reduced glucose tolerance [29, 30]. In our study, a high level of self-reported physical activity was significantly more common in the NGT group, even after BMI adjustment, and correlated negatively with waist circumference, HOMA-IR, fasting insulin and glucose, and HbA1c, suggesting a strong preventive or delaying effect of IGT. Thus, to identify women at high risk for diabetes after GDM is critical for individual risk stratification and early prevention after delivery (e.g., with increased physical activity).

With the availability and advancement of methods such as NMR and mass spectrometry, several other nonglucose metabolites associated with insulin resistance have been identified, such as 2-hydroxybutyrate (interestingly also linked to amino acid metabolism and the TCA cycle in similarity with 3-HIB), lipid signalling molecules, and fatty acids [31, 32]. The identification of BCAAs, 3-HIB, and these other metabolites in insulin resistance might provide further information in mechanistic studies of metabolic disease, as well as provide different combinations of markers for early prediction of disease and for evaluation of therapeutic interventions.

A limitation of the study is a potential recruitment bias out of the total Gothenburg GDM population, possibly due to language difficulties in the non-Nordic ethnic population. However, this factor is not likely to affect comparisons between glucose tolerance groups or correlations of metabolic parameters.

## 5. Conclusion

Metabolic risk scores that can identify women at the highest risk for transitioning from GDM to IGT and T2DM are needed. A cost-effective and easy-to-use strategy must be adopted by healthcare givers to reach these women and offer individualized prevention programs. A single blood sample taken annually to analyse one or a few metabolites, in addition to well-known clinical risk factors, would be beneficial but is currently unavailable. Thus, 3-HIB is an interesting metabolite warranting further evaluation as one of a suite of markers for insulin resistance beyond the risk associated with obesity and might improve the prediction of future metabolic risk.

## Figures and Tables

**Table 1:** Anthropometry, blood pressure (BP), clinical blood measurements, and self-reported lifestyle data at follow-up 6 years after GDM pregnancy.

Variable		NGT			IGT			T2DM		*P*			*P* (BMI adjusted)		
	*n*	Mean	SD	*n*	Mean	SD	*n*	Mean	SD	IGT versus NGT	T2D versus NGT	T2D versus IGT	IGT versus NGT	T2D versus NGT	T2D versus IGT
*Anthropometry and blood pressure*															
BMI (kg/m^2^)	139	26.1	4.8	46	29.4	4.5	44	29.1	5.8	<0.001	0.002				
BMI change (kg/m^2^)	139	−0.4	3.0	46	1.8	3.1	44	−0.4	3.5	<0.001		0.003	0.001		
Waist (cm)	137	87.5	10.8	45	94.2	16.2	44	94.6	14.5	0.007	0.004				
Hip (cm)	137	102.3	9.7	45	108.4	9.1	44	107.1	11.8	0.002	0.02				
Waist : hip ratio	137	0.9	0.1	45	0.9	0.1	44	0.9	0.1						
Body fat (%)	59	32.9	7.9	16	40.5	6.6	12	38.5	13.2	0.007					
Fat mass (kg)	59	23.4	9.5	16	32.3	10.7	12	31.8	15.8	0.01	0.04				
Fat-free mass (kg)	59	45.6	5.4	16	46.0	6.7	12	45.9	6.0						
BP systolic (mmHg)	138	113.9	12.8	46	122.9	14.0	44	119.2	14.2	<0.001			0.02		
BP diastolic (mmHg)	138	74.9	9.6	46	78.6	9.8	44	78.1	10.6						
*Clinical blood measurements*															
b-HbA1c (mmol/mol)	139	37.1	3.7	45	38.4	3.8	44	52.6	18.8		<0.001	<0.001		<0.001	<0.001
p-glucose fasting (mM)	138	5.4	0.4	43	6.1	0.4	44	8.2	3.5	0.02	<0.001	<0.001	0.02	<0.001	<0.001
p-glucose 2 h (mM)	128	5.5	1.1	42	7.8	1.6	12	11.8	3.9	<0.001	<0.001	<0.001	<0.001	<0.001	<0.001
s-insulin fasting (mU/L)	134	8.0	4.2	43	14.4	9.5	44	12.8	7.4	<0.001	<0.001		<0.001	0.003	
s-insulin 2 h (mU/L)	121	44.7	37.7	40	87.1	57.1	12	85.3	58.4	<0.001	0.008		<0.001	0.01	
HOMA-IR	133	1.9	1.0	43	3.8	2.7	43	4.9	3.4	<0.001	<0.001	0.04	<0.001	<0.001	0.004
s-total cholesterol (mM)	137	4.7	0.8	45	4.7	0.8	43	4.7	0.7						
s-HDL (mM)	138	1.5	0.3	45	1.3	0.3	44	1.4	0.4	0.001	0.04		0.04		
s-LDL (mM)	138	2.9	0.8	45	3.1	0.7	44	3.0	0.6						
s-triglycerides (mM)	137	0.9	0.5	45	1.2	0.5	44	1.4	1.4		<0.001			0.002	
s-leptin (mg/L)	134	18.2	11.8	42	29.3	12.9	44	24.4	22.4	<0.001	0.04		0.03		
s-adiponectin (ug/L)	137	17.8	14.5	41	18.1	15.9	44	14.2	12.9						
Leptin : adiponectin 10^−3^	137	1.86	1.95	41	2.83	2.52	44	3.51	3.67		0.001			0.02	
s-C-reactive protein (mg/L)	137	2.1	3.6	43	3.1	3.1	44	5.5	8.2		<0.001			0.008	0.007
p-free fatty acids (mM)	138	0.43	0.18	41	0.56	0.20	44	0.59	0.22	0.001	<0.001		0.001	<0.001	
ADIPO-IR (mM·pM)	134	24.1	17.6	40	53.6	38.2	44	55.7	40.5	<0.001	<0.001		<0.001	<0.001	
*Questionnaires*															
Physical activity	120	4.0	1.1	38	3.3	1.0	34	3.4	1.3	0.008	0.02		0.009	0.02	
Energy intake (kcal)	88	2390	561	31	2366	735	17	2258	610						
Protein intake (g/kg)	88	1.4	0.4	31	1.2	0.4	17	1.4	0.5						
Fat intake (g/kg)	88	1.6	0.5	31	1.3	0.4	17	1.5	0.5						
Carbohydrate intake (g/kg)	88	3.7	1.2	31	3.2	1.3	17	3.4	1.3						

The women were divided into glucose tolerance groups based on follow-up OGTT. Statistical analyses were done with ANOVA (*P*) and ANCOVA (BMI-adjusted *P*). BMI change refers to change from start of pregnancy to follow-up. Macronutrient intake expressed as g/kg body weight. b, whole blood; p, plasma; s, serum.

**Table 2:** NMR metabolites at follow-up 6 years after GDM pregnancy.

%	NGT		IGT		T2DM		*P*			*P* (BMI adjusted)		
	Mean	SD	Mean	SD	Mean	SD	IGT versus NGT	T2D versus NGT	T2D versus IGT	IGT versus NGT	T2D versus NGT	T2D versus IGT
Phenylalanine	100.0	45.4	121.8	43.8	117.1	49.1	0.02					
Tyrosine	100.0	25.1	109.6	26.9	111.6	26.7		0.03				
Glucose	100.0	20.7	112.1	18.1	159.5	79.6		<0.001	<0.001		<0.001	<0.001
Mannose	100.0	25.9	113.4	26.2	144.3	59.5		<0.001	<0.001		<0.001	<0.001
Glycerol	100.0	43.1	127.5	48.6	140.7	71.1	0.006	<0.001		0.007	<0.001	
Glycine	100.0	27.0	88.1	19.1	89.6	25.6	0.02	0.05		0.04		
Citrate	100.0	31.0	111.5	23.9	117.3	25.7		0.002			0.002	
Pyruvate	100.0	80.6	141.3	78.2	119.4	73.7	0.008			0.01		
Acetoacetate	100.0	105.8	115.7	103.9	253.1	348.8		<0.001	0.001		<0.001	0.001
3-HIB	100.0	54.7	128.2	52.7	131.5	75.5	0.02	0.006		0.005	0.002	
Valine	100.0	19.2	108.2	15.1	109.5	21.6	0.04	0.01			0.05	
Isoleucine	100.0	22.1	109.0	19.0	115.7	28.5		<0.001			0.001	
Leucine	100.0	18.2	111.2	13.6	113.4	22.7	0.002	<0.001		0.01	0.001	

Metabolites significantly different between groups are shown. Data is expressed as % of NGT. Statistics are performed using ANOVA (*P*) and ANCOVA (BMI-adjusted *P*). *n* = 137 for NGT, *n* = 43 for IGT, and *n* = 44 for T2DM.

**Table 3:** Stepwise linear regression model with HOMA-IR as dependent variable and follow-up variables as independent variables.

Variables included in model	*β*	*P*
s-triglycerides (mM)	0.35	<0.001
Waist (cm)	0.31	<0.001
3-HIB (%)	0.19	<0.001
Age (y)	−0.18	<0.001
s-C-reactive protein (mg/L)	0.15	0.008
Diastolic BP (mmHg)	0.13	0.01
Physical activity	−0.12	0.01
BMI change (kg/m^2^)	0.11	0.02

Out of 22 variables, 8 remained in the final model to predict HOMA-IR variance; *R* = 0.80, *R* ^2^ = 0.64, and adjusted *R* ^2^ = 0.62.

## Data Availability

Data can be provided on request.

## Abbreviations

3-HIB: 3-Hydroxyisobutyrate
AA: Amino acid
AAA: Aromatic amino acid
ADIPO-IR: Adipose tissue insulin resistance
BCAA: Branched-chain amino acid
BMR: Basic metabolic rate
EI: Energy intake
FFA: Free fatty acids
GDM: Gestational diabetes mellitus
IGT: Impaired glucose tolerance
NGT: Normal glucose tolerance
OGTT: Oral glucose tolerance test
T2DM: Type 2 diabetes mellitus.

## Supplementary Materials

Supplementary Materials NMR data supporting annotation of methyl signal at 1.0679 ppm as 3-HIB. TOCSY cross peaks at 2.483, 3.535, and 3.703 ppm are in accordance with the 3-HIB HMDB entry 00023 values of 2.4727, 3.5244, and 3.6844. There is also a very weak signal in the natural abundance of 1H,13C-HSQC at a carbon chemical shift of 16.59 ppm, also in accordance with the 3-HIB methyl carbon shift in HMDB of 16.60 ppm.
