# Supplementary material for: Higher Concentrations of BCAAs and 3-HIB Are Associated with Insulin Resistance in the Transition from Gestational Diabetes to Type 2 Diabetes
Source: J Diabetes Res. 2018 Jun 5;2018:4207067. doi: 10.1155/2018/4207067 (PMC6008749; doi:10.1155/2018/4207067)
Supplement: Supplementary Materials — NMR data supporting annotation of methyl signal at 1.0679 ppm as 3-HIB. TOCSY cross peaks at 2.483, 3.535, and 3.703 ppm are in accordance with the 3-HIB HMDB entry 00023 values of 2.4727, 3.5244, and 3.6844. There is also a very weak signal in the natural abundance of 1H,13C-HSQC at a carbon chemical shift of 16.59 ppm, also in accordance with the 3-HIB methyl carbon shift in HMDB of 16.60 ppm. [file 4207067.f1.pptx]

## Slide 1
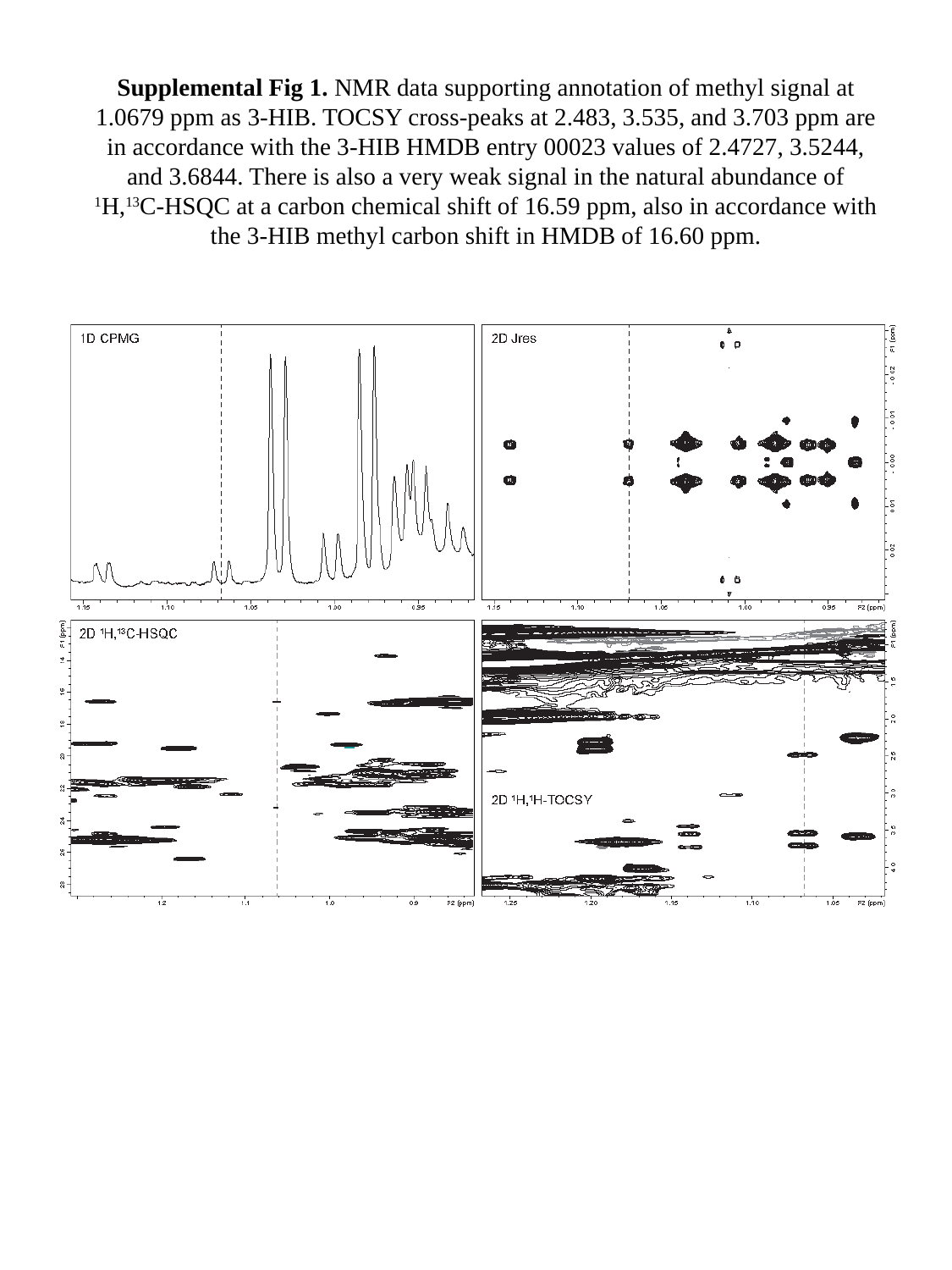

Supplemental Fig 1. NMR data supporting annotation of methyl signal at 1.0679 ppm as 3-HIB. TOCSY cross-peaks at 2.483, 3.535, and 3.703 ppm are in accordance with the 3-HIB HMDB entry 00023 values of 2.4727, 3.5244, and 3.6844. There is also a very weak signal in the natural abundance of 1H,13C-HSQC at a carbon chemical shift of 16.59 ppm, also in accordance with the 3-HIB methyl carbon shift in HMDB of 16.60 ppm.
